# Bilateral Vitreopapillary Traction Demonstrated by Optical Coherence Tomography Mistaken for Papilledema

**DOI:** 10.1155/2012/682659

**Published:** 2012-10-04

**Authors:** Elizabeth Houle, Neil R. Miller

**Affiliations:** ^1^Johns Hopkins University School of Medicine, Baltimore, MD 21205-2196, USA; ^2^Wilmer Eye Institute, Johns Hopkins Hospital, Woods 458, 600 N Wolfe Street, Baltimore, MD 21287, USA

## Abstract

*Purpose*. The purpose of this study was to report a case of bilateral vitreopapillary traction, previously misdiagnosed as papilledema. *Methods*. A case report is presented of a 47-year-old woman with a prior diagnosis of papilledema, who is shown to have bilateral vitreopapillary traction rather than true optic disc swelling, confirmed by optical coherence tomography (OCT). *Results*. OCT showed vitreous traction surrounding the optic discs of both eyes. Fluorescein angiography demonstrated focal leakage of both discs. *Conclusion*. Bilateral disc elevation caused by vitreous traction can be confused with papilledema. In such cases, OCT can be used to arrive at the correct diagnosis. Although the phenomenon of vitreopapillary traction is well reported, this case indicates that not all ophthalmologists recognize the condition.

## 1. Introduction

Vitreopapillary traction (optic disc traction) is characterized by traction of the optic disc by a fibrocellular proliferating membrane or an incomplete posterior vitreous detachment [1]. It has been described in the context of diabetic retinopathy and has been observed in association with central retinal vein occlusion and nonarteritic anterior ischemic optic neuropathy, suggesting a possible contributing factor to these disorders [2–4]. When the vitreous exerts traction on the optic nerve head, it can lead to elevation of the disc, obscuration of the disc margins, peripapillary hemorrhage, and even some disc leakage on fluorescein angiography, thus simulating true optic disc swelling.

## 2. Report of a Case

A 47-year-old woman with mild hypercholesterolemia was referred to the Neuro-Ophthalmology Division of the Wilmer Eye Institute for evaluation of bilateral optic disc swelling. Two years earlier, she had complained of bilateral blurred vision and was noted to have bilateral optic disc elevation. Computed tomographic scanning, magnetic resonance (MR) imaging, and MR venography were reported to show no abnormalities. The patient was thought to have pseudotumor cerebri, started on acetazolamide, and referred to a neurologist. A year later, she underwent lumbar puncture that demonstrated an opening pressure of 18 mm H_2_O with normal cerebrospinal fluid content. She subsequently discontinued acetazolamide and presented to our clinic 1 year later because of persistent mild blurred vision. She denied a history of headache, transient visual obscurations, or tinnitus. Her only medication was an oral contraceptive agent.

**Figure 1:**
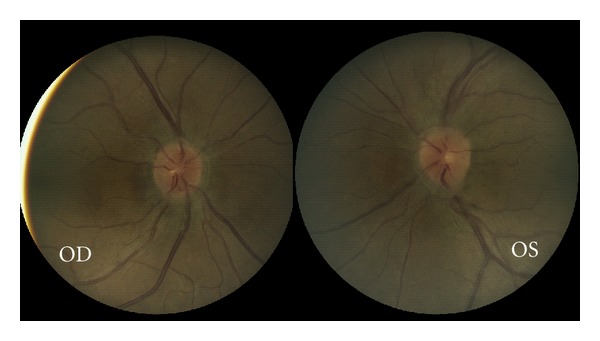
Color fundus photographs of the right and left optic nerves.

On examination, the patient had a body mass index of 26.6. Her visual acuity was 20/20 in both eyes with normal color vision and full visual fields by automated perimetry. Pupils were briskly and equally reactive with no relative afferent pupillary defect. Ocular motility and alignment were normal. Cranial nerves five, seven, and eight were intact. Intraocular pressures were normal, and slit-lamp examination revealed a normal anterior segment. Ophthalmoscopic examination revealed slight elevation of both optic discs, associated with a ring of vitreous traction obscuring the retinal vessels (Figure 1). There were no retinal hemorrhages or exudates. The maculae were flat, and the retinal vessels and peripheral retina showed no abnormalities.

**Figure 2:**
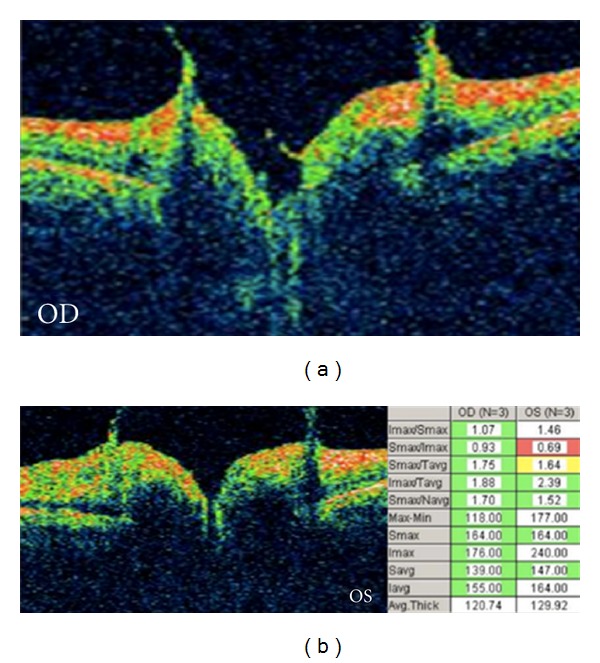
Optical coherence tomography of the right and left optic nerves demonstrating bilateral vitreopapillary traction.

**Figure 3:**
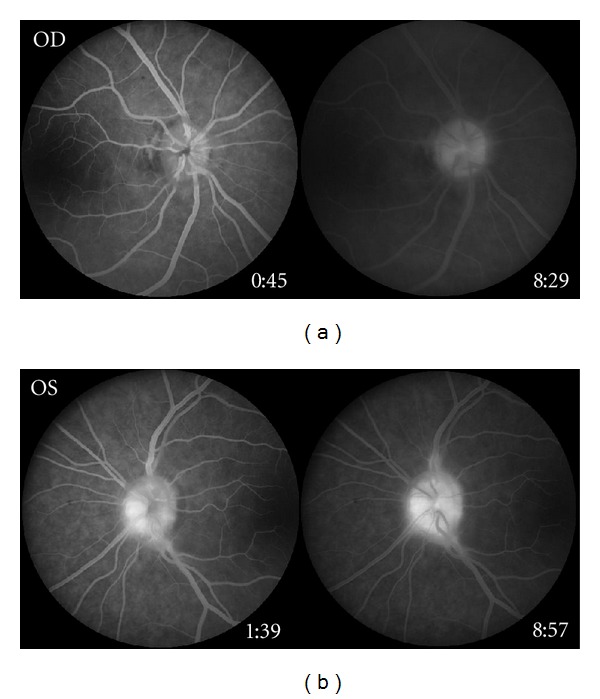
(a) Fluorescein angiogram of the right optic disc demonstrating focal leakage. (b) Fluorescein angiogram of the left optic disc demonstrating focal leakage.

OCT showed moderate increased thickness of the peripapillary retinal nerve fiber layer and vitreous traction surrounding the optic discs of both eyes (Figure 2). Fluorescein angiography demonstrated focal leakage of both discs (Figures 3(a) and 3(b)).

## 3. Comment

Posterior vitreous detachment occurs when the vitreous liquefies with age and the posterior vitreous cortex separates from the internal limiting membrane. Recent evidence suggests that posterior vitreous detachment originates in the perifoveal region and progresses gradually over a course of months to years, with the vitreous eventually detaching from the optic nerve head [5]. Vitreopapillary traction occurs when an anomalous or partial posterior vitreous detachment or fibrocellular membrane exerts traction on the optic nerve head [3]. Vitreopapillary traction can produce intrapapillary and peripapillary hemorrhage and can cause elevation of the optic discs which has been documented by both ultrasonography [6] and OCT [7]. Indeed, Nomura et al. described a patient with an altitudinal visual field deficit in whom spectral domain OCT demonstrated a corresponding area of sectoral vitreopapillary traction [8]. Our case illustrates that bilateral disc elevation caused by vitreous traction can be confused with papilledema, but that in such cases, OCT can be used to arrive at the correct diagnosis. Although the phenomenon of vitreopapillary traction is well reported, this case indicates that not all ophthalmologists recognize the condition.
